# Guanine-rich sequences inhibit proofreading DNA polymerases

**DOI:** 10.1038/srep28769

**Published:** 2016-06-28

**Authors:** Xiao-Jing Zhu, Shuhui Sun, Binghua Xie, Xuemei Hu, Zunyi Zhang, Mengsheng Qiu, Zhong-Min Dai

**Affiliations:** 1 Institute of Life Sciences, Key Laboratory of Organ Development and Regeneration of Zhejiang Province, College of Life Sciences, Hangzhou Normal University, Hangzhou, Zhejiang, 310036, PR China; 2 Department of Anatomical Sciences and Neurobiology, University of Louisville, Louisville, KY40292, USA

## Abstract

DNA polymerases with proofreading activity are important for accurate amplification of target DNA. Despite numerous efforts have been made to improve the proofreading DNA polymerases, they are more susceptible to be failed in PCR than non-proofreading DNA polymerases. Here we showed that proofreading DNA polymerases can be inhibited by certain primers. Further analysis showed that G-rich sequences such as GGGGG and GGGGHGG can cause PCR failure using proofreading DNA polymerases but not Taq DNA polymerase. The inhibitory effect of these G-rich sequences is caused by G-quadruplex and is dose dependent. G-rich inhibitory sequence-containing primers can be used in PCR at a lower concentration to amplify its target DNA fragment.

Polymerase chain reaction (PCR) is now a routine technique used in molecular biology^1^,^2^. PCR is so powerful that an increasing number of PCR-related techniques have been developed^3^. And numerous thermostable DNA polymerase with distinct properties were characterized and engineered to improve PCR performance and extend PCR application^4^,^5^. Initially, PCR can be only used to amplify short DNA fragments. Because of the high demand for accurate amplification of long products, great efforts have been made to extend the range for PCR. The most effective method for long PCR was achieved by DNA polymerase blend, composed by adding a small amount of proofreading (3′ → 5′ exonuclease) DNA polymerase into a non-proofreading DNA polymerase^6^,^7^. Another method allowing the amplification of very long DNA fragment is fusing DNA polymerase with a non-specific double strand DNA binding protein Sso7d, which also dramatically increase the processivity and fidelity of DNA polymerase^8^.

Here we compared the performance of several different commercial available polymerases. We found that engineered proofreading (high fidelity) DNA polymerases are good and sometimes even better than DNA polymerase blend. However, we still faced some problem in PCR using proofreading DNA polymerases. To our surprise, we found that during PCR, proofreading DNA polymerases can be inhibited by G-rich sequences. This inhibitory effect is different from the previous described aptamers, as the latter ones only inhibit polymerase at lower temperature^9^,^10^,^11^.

## Results

### Engineered proofreading DNA polymerases are greatly enhanced

**Figure 1:**
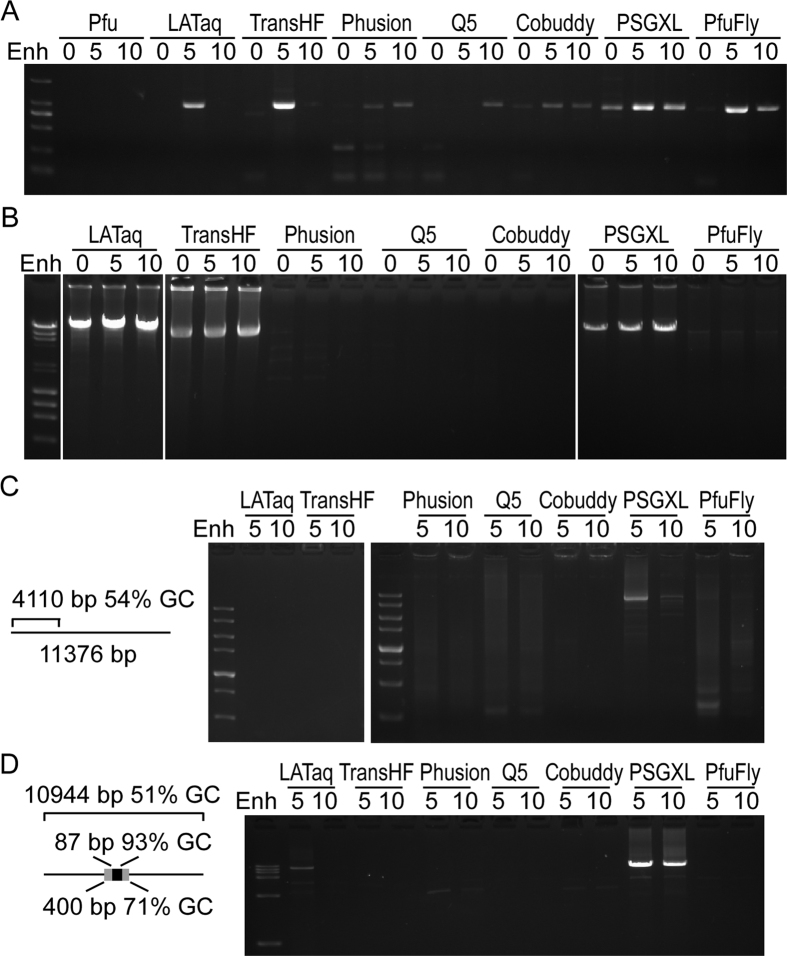
Engineered proofreading DNA polymerases have good performance. (**A**) Testing various DNA polymerases for the amplification of a 1 kb fragment with 70% GC-content. (**B**) Testing different DNA polymerases for the amplification of an 18 kb fragment from λ DNA. (**C**) Amplification of the ORF of insulin-like growth factor I receptor (Igf1r) from mouse cDNA. The 3′ untranslated region of Igf1r is longer than 7 kb. (**D**) Amplification of the entire LCas9 plasmid. The 10.9 kb LCas9 plasmid contains a high GC-rich region. Pfu: Pfu DNA polymerase. LATaq: LA Taq Version 2.0. TransHF: TransTaq DNA polymerase High Fidelity. Phusion: Phusion High-Fidelity DNA polymerase. Q5: Q5 High-Fidelity DNA polymerase. Cobuddy: Cobuddy Super Fidelity DNA polymerase. PSGXL: PrimeSTAR GXL DNA polymerase. PfuFly: TransStart FastPfu Fly DNA polymerase. Enh: 0–10 μl of GC enhancer was added into the 25 μl PCR reagent.

To find out optimal DNA polymerase for accurate amplification of long and GC-rich product, we compared several types of thermostable DNA polymerases. We firstly tested the enzymes to amplify a 1 kb fragment with high GC-contents (70%). All the tested enzymes except Pfu are able to amplify the GC-rich fragment efficiently (Fig. 1A). The results showed that the yield of PCR for all enzymes except Q5 increased more with 5 μl of GC enhancer than 10 μl of GC enhancer (Fig. 1A). We next test the amplification of an 18 kb fragment with slightly high GC-contents (57%) from λ phage DNA. Although all the tested enzymes are claimed by their manufacturer to be able to efficiently amplify fragment longer than 20 kb from λ phage DNA, only LATaq, TransHF and PSGXL showed a robust amplification of the 18 kb fragment (Fig. 1B). A weak amplification of the 18 kb fragment was detected by using Phusion and PfuFly but not Q5 and Cobuddy (Fig. 1B). Our results also suggested that adding 5 μl of the GC enhancer improves the PCR efficiency (Fig. 1A,B). We then test the amplification of the open reading frame (ORF) of Igf1r genes from a more complex cDNA sample. LATaq, TransHF, Phusion and Cobuddy failed to amplify the target. However, a weak target band within a smear was observed by using Q5 and PfuFly. And the best amplification was obtained by PSGXL, although with several nonspecific bands (Fig. 1C). We finally test these enzymes for the PCR amplification of an LCas9 plasmid. The GC-contents of the entire plasmid is moderate (51%), but there is a highly GC-rich region in this plasmid (an 87 bp of 93% GC-contents within a 400 bp of 71% GC-contents). Only LATaq and PSGXL can efficiently amplify the entire plasmid (Fig. 1D). These results showed that the engineered proofreading DNA polymerases are reasonable good for long and accurate PCR.

### Primers inhibit proofreading DNA polymerases during PCR

**Figure 2:**
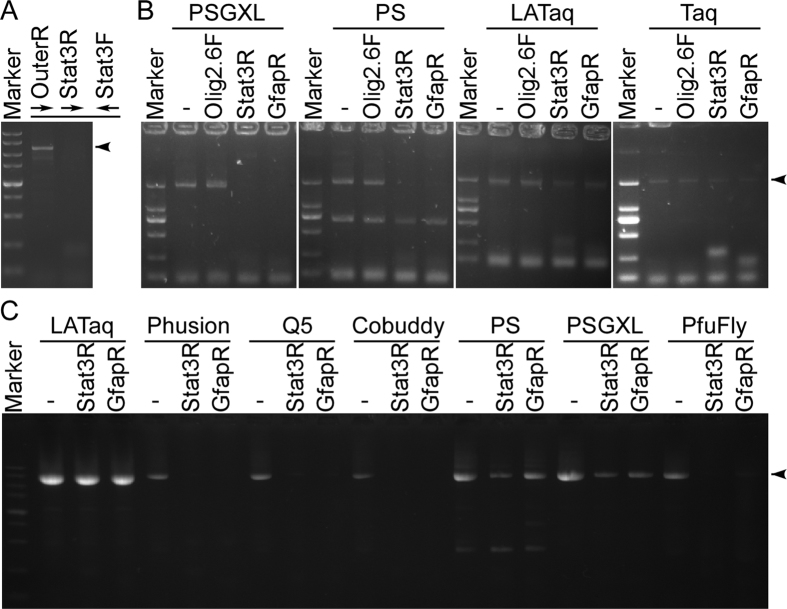
Proofreading DNA polymerases can be inhibited by certain primers. (**A**) Example of inhibitory primer using PSGXL. A slightly longer target DNA fragment can be successfully amplified by the primers OuterR and Stat3F, but use Stat3R instead of OuterR caused PCR failure. (**B**) Primers Stat3R and GfapR are inhibitory to PSGXL and PS, but not to LATaq and Taq. The 2 kb targets were amplified using primers Olig2F and Olig2R, additional primer Olig2.6F, Stat3R, GfapR were added to the reaction. (**C**) Primers Stat3R and GfapR are inhibitory to all the tested proofreading DNA polymerases. Primers pBSIIF and pBSIIR were used to amplify the entire plasmid pBlueScript II KS (−). Adding the additional primer Stat3R or GfapR substantially reduced the PCR yield using the proofreading DNA polymerases such as Phusion, Q5, Cobuddy, PS, PSGXL and PfuFly. Arrowheads indicate target DNA fragments.

Although PSGXL is robust to amplify targets in most cases, we always failed to amplify the target when the primer Stat3R or GfapR (all the oligonucleotides are listed in Table 1) is used. For example, we failed to amplify the ORF of the Stat3 gene using Stat3F and Stat3R, but substitute the Stat3R by an outer primer OuterR allows us to amplify the Stat3 ORF-containing DNA fragment efficiently (Fig. 2A). We still failed to amplify the Stat3 ORF with the primers Stat3F and Stat3R even though nested PCR were used (data not shown). We proposed that the primers Stat3R and GfapR may possess an inhibitory effect during PCR.

To examine if PSGXL is inhibited by Stat3R and GfapR, we additional added one of the two primers in PCR to compare the yield of target. Our result showed that a 2 kb mouse genomic DNA fragment can be successfully amplified by primers Olig2F and Olig2R, and the additional primer Olig2.6F did not inhibit the amplification. As expected, both Stat3R and GfapR can inhibit the amplification (Fig. 2B). As PSGXL is processivity-enhanced version of PS, we next tested that if the primers Stat3R and GfapR can also inhibit PS. Our results showed that the primers Stat3R and GfapR also showed an inhibitory effect to PS (Fig. 2B), indicating that the primers inhibit the DNA polymerase directly rather than inhibit the processivity-enhancing factor. We also tested to see if the two primers inhibit other DNA polymerases. In contrast to PS and PSGXL, Taq and LATaq were not inhibited by the primers Stat3R and GfapR (Fig. 2B). We then asked that if the primers Stat3R and GfapR inhibit other proofreading DNA polymerases. For this purpose, we used a different primer pair pBSF and pBSR to amplify the entire plasmid pBlueScript II KS (−). Consistently LATaq was not inhibited by the primers Stat3R and GfapR, whereas all the tested proofreading DNA polymerases such as Phusion, Q5, Cobuddy, PS, PSGXL and PfuFly were substantially inhibited by the primers Stat3R and GfapR (Fig. 2C). Taken together, these results demonstrated that primers Stat3R and GfapR can inhibit the proofreading DNA polymerases during PCR.

### G-rich sequences caused inhibitory effect

**Figure 3:**
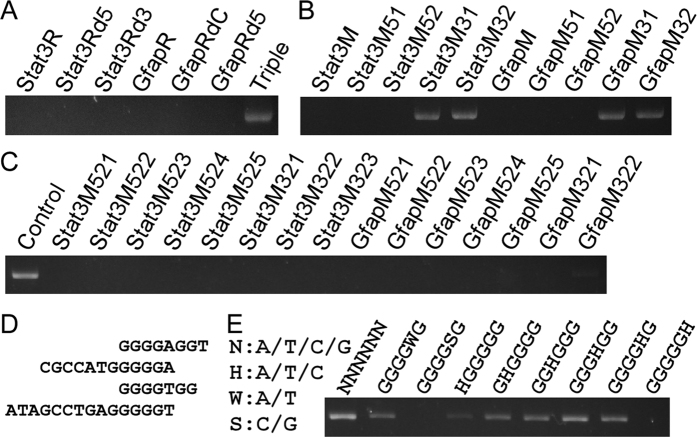
G-rich sequences caused PCR inhibitory effect. (**A**) Deletion of CGCAGATC sequence from Stat3R and GfapR remains their inhibitory effect. (**B,C**) The sequentially terminal truncated forms of Stat3R and GfapR were used to test their inhibitory effect. (**D**) The shortest inhibitory sequences from **B,C**. (**E**) GGGGGH is sufficient to inhibit PCR using proofreading DNA polymerase.

To determine the minimum sequence causing the inhibitory effect to the proofreading DNA polymerases, we compared the primers Stat3R and GfapR and found that both primers contain a sequence of CGCAGATC. We therefore examined four primers Stat3Rd5, GfapRdC, Stat3Rd3 and GfapRd5 to see if they can inhibit PCR. The former two primers are CGCAGATC-deleted forms of the primers Stat3R and GfapR respectively, whereas the latter two are generated by other deletion (Table 1). Contrary to our expectation, the result showed that all the four tested primers can strongly inhibit the PCR amplification (Fig. 3A). And the CGCAGATC-triplicated primer Triple showed no inhibitory effect (Fig. 3A). The results demonstrated that the inhibitory effect is independent of CGCAGATC sequence.

As we have obtained insufficient information from sequence analysis, we switched to determine when the primers lose their inhibitory effect by shortening a few bases each time. The primer Stat3M consists of the overlapped sequences from Stat3Rd5 and Stat3Rd3, and the primer GfapM is derived from the overlapped region of GfapRdC and GfapRd5 (Table 1). Primers with 4–8 bases deletion at the 5′ terminal of Stat3M and GfapM still inhibited the PCR amplification, but 4 bases deletion at the 3′ terminal of Stat3M and GfapM diminished their inhibitory effect (Fig. 3B). We then further checked primers with various deletion at the ends. This narrowed down the inhibitory sequences to four short primers (Fig. 3C,D). All the four short primers contain G-rich sequences (Fig. 3D). To find out the exact inhibitory sequences, we tested various G-rich hexamers. Compared with random hexamer, the band is weaker when any of the G-rich hexamer was added to PCR (Fig. 3E). Target amplification was strongly inhibited by the HGGGGG hexamer, and thoroughly inhibited by the hexamers with the sequence of GGGGSG and GGGGGH, suggesting that five consecutive G is sufficient to inhibit PCR using proofreading DNA polymerases (Fig. 3E). Taken together, these results indicated that sequences such as GGGGG and GGGGNGG cause inhibitory effect to the proofreading DNA polymerases.

### Decrease the inhibitory effect by reducing the dose of inhibitory sequence

**Figure 4:**
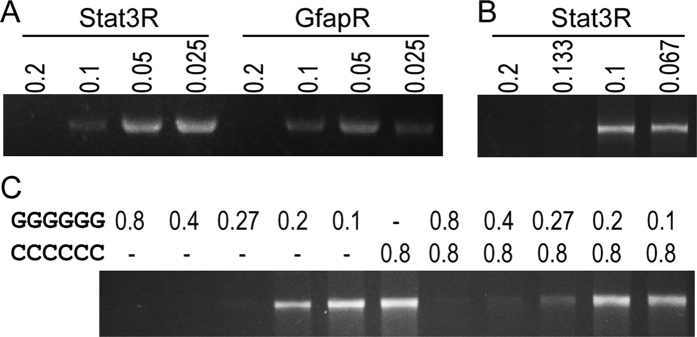
The inhibitory effects of the G-rich primers are tunable. (**A**) Dose-dependent inhibitory effect of G-rich oligonucleotides. Primers Olig2F and Olig2R were used to amplify their 2 kb target. The inhibitory effect of the additional oligonucleotides Stat3R and GfapR were diminished when their final concentration is below 0.1 μM. (**B**) Lowering the final concentration of the inhibitory primer enables it to amplify its target. Stat3F and various amount of Stat3R were used to amplify their target. Only when the Stat3R were used at concentration of 0.133 μM or below can the target be amplified efficiently. (**C**) Inhibitory effect of G-rich sequences can be reduced by their complementary sequences. Additional oligonucleotides GGGGGG and CCCCCC were added to see the yield of the PCR products using Olig2F and Olig2R as primers.

We used a serial dilution of the primers Stat3R and GfapR to examine if these G-containing oligonucleotides inhibit PCR in a dose-dependent manner. Primers Stat3R and GfapR still caused PCR failure at the concentration of 0.2 μM, but the target products were increased when their concentration is equal to or below 0.1 μM (Fig. 4A). This indicated that the inhibitory effect is dose-dependent. Can an inhibitory primer be used at lower concentrations that its inhibitory effect is greatly reduced but its priming efficiency remains high? To answer this question, we used the primers Stat3F and Stat3R to amplify the ORF of Stat3. Our results showed that the target band becomes visible when the concentration of Stat3R decreasing from 0.2 μM to 0.133 μM, and the amplification efficiency is further increased when the concentration of Stat3R was used at 0.1 μM and 0.067 μM (Fig. 4B). This suggested that the amplification efficiency could be increased when the amount of inhibitory primer is decreased to reduce its inhibitory effect. We then raise a question that if adding an oligonucleotide complementary to the inhibitory sequences could reduce the inhibitory effect. To test this, we used hexamer GGGGGG and its complement CCCCCC. Our result showed that without CCCCCC, target bands were visible when the concentration of GGGGGG is equal to or below 0.27 μM, whereas in the presence of CCCCCC, target bands were visible even the concentration of GGGGGG is as high as 0.8 μM (Fig. 4C). This suggested that the inhibitory effect of the G-rich sequences can be reduced by adding complementary oligonucleotides.

### Proofreading DNA polymerases specifically bind to G-quadruplex

**Figure 5:**
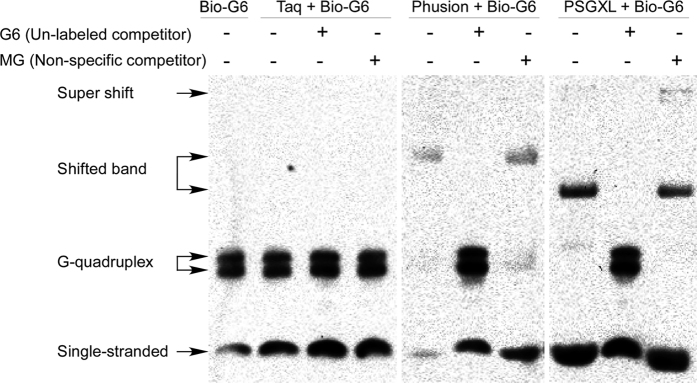
Proofreading DNA polymerases but not Taq DNA polymerase bind to G-rich sequences. Biotin-labeled oligonucleotide Bio-G6 was subjected to electrophoresis mobility shift assay. Electrophoresis of the Bio-G6 alone showed 3 bands in the gel, as the 6 consecutive G in Bio-G6 tends to form intermolecular G-quadruplex. Taq didn’t cause any band shift. However, when the proofreading DNA polymerase Phusion or PSGXL was added, the bands corresponding to intermolecular G-quadruplex were retarded. The band shift caused by proofreading DNA polymerases were disappeared by adding excess amount of competitor G6 but not non-specific competitor MG. Note that the anti-PSGXL antibody also caused a weak supershift.

As secondary structure of the G-rich oligonucleotides are normally disrupted during PCR, and the inhibitory effect of G-rich sequences could be reduced by adding its complementary oligonucleotide (Fig. 4), one may concluded that the inhibition of proofreading DNA polymerases is caused by single-stranded G-rich sequences. However, G-rich sequences tends to form G-quadruplex structure^12^, and the possibility of forming intermolecular G-quadruplex is also decreased by lowering the concentration of single-stranded G-rich oligonucleotides. To investigate how G-rich sequences inhibit the proofreading DNA polymerases, we performed electrophoresis mobility assay (EMSA). Electrophoresis of Bio-G6 (Table 1) showed that there are two additional bands, which represent the intermolecular G-quadruplex (Fig. 5). Taq didn’t cause any shift of the Bio-G6 bands (Fig. 5), indicating that the affinity between Taq and G-rich sequences is too low to be detected. However, the G-quadruplex bands of Bio-G6 were shifted when there is proofreading DNA polymerase Phusion or PSGXL. The shifted bands disappeared and the G-quadruplex bands reappeared when excess amount of G6 was added as unlabeled specific competitor (Fig. 5). However, the shifted bands persisted even there was 1000 excess amount of MG (mutated from G6) as non-specific competitor, indicating that proofreading DNA polymerases specifically bind to G-quadruplex.

## Discussion

The use of proofreading DNA polymerases to amplify target DNA fragments is highly demanded for cloning of targets without undesired mutations. Although great efforts have been made to engineer the proofreading DNA polymerase to enhance both their fidelity and performance^4^,^5^,^8^, we still failed to amplify some target using proofreading DNA polymerases for unknown reason. Here we showed that oligonucleotides containing G-rich sequences such as GGGGG and GGGGHGG can inhibit proofreading DNA polymerases in a dose-dependent manner.

The inhibition effect to proofreading DNA polymerases is not significant when the G-rich primer is used below 0.1 μM (Fig. 4). As the Sso7d fused proofreading DNA polymerases such as Phusion, Q5 and Cobuddy are recommended to use primers at a final concentration of 0.5 μM, PCR should be failed if a G-rich primer was used. If a PCR is inefficient to amplify the target, researchers may try to enhance the amplification efficiency by adding more primers to increase the template-primer binding. This will further reduce the efficiency of PCR if proofreading DNA polymerases and G-rich primers are used. In such cases, use the inhibitory primers at lower concentrations such as 0.1 μM and 0.067 μM will greatly improve the PCR yield (Fig. 4B).

The inhibitory sequences such as GGGGG and GGGGHGG may form intermolecular G-quadruplex structure, a structure that may interfere DNA synthesis at physiological conditions^12^,^13^. And some G-rich aptamers, which can form intramolecular G-quadruplex structure, can inhibit Taq and some other DNA polymerases^10^,^11^. These aptamers only showed inhibitory effect at low temperature, and the inhibitory effect disappeared as the G-quadruplex structure is disrupted during the annealing and extension procedure of PCR. The low temperature-dependent inhibitory effect allows these aptamers be used in PCR to amplify target more specifically and efficiently^11^. However, the G-rich inhibitory oligonucleotides described here strongly inhibited the proofreading DNA polymerases even during the PCR cycling procedure. Previously, it has been revealed that intermolecular G-quadruplex is stable^14^, especially in potassium-containing buffers. Our results showed that Phusion and PSGXL specifically bind to G-quadruplex formed by G-rich sequences (Fig. 5), suggesting that it is the G-quadruplex but not the single-stranded form of G-rich sequences binds to and inhibits the proofreading DNA polymerases. Further studies may required to investigate the mechanism of the interaction of proofreading DNA polymerase and G-quadruplex, which may help us understand the archaeal genome replication, and may also help us to modify the proofreading DNA polymerases to improve their performance.

## Methods

### Thermostable DNA polymerases

The following thermostable DNA polymerases were used in this study. Taq DNA polymerase (Taq) and Pfu DNA polymerase (Pfu) are purchased from Sangon Biotech (Shanghai, China). LA Taq™ Version 2.0 (LATaq), PrimeSTAR (PS) and PrimeSTAR^®^ GXL DNA Polymerase (PSGXL) are from TaKaRa Bio (Dalian, China). TransTaq DNA Polymerase High Fidelity (TransHF) and TransStart FastPfu Fly DNA Polymerase (PfuFly) are from TransGen Biotech (Beijing, China). Phusion^®^ High-Fidelity DNA Polymerase (Phusion) and Q5^®^ High-Fidelity DNA Polymerase (Q5) are from New England Biolabs. Cobuddy Super Fidelity DNA Polymerase (Cobuddy) is purchase from CWBiotech (Beijing, China).

We choose the above described polymerases based on their availability as well as they represent different properties. Taq belongs to family A DNA polymerase which lacks proofreading activity^4^,^5^. Whereas the family B DNA polymerases Pfu and PS have much higher fidelity than Taq because of their proofreading (3′ → 5′ exonuclease) activity, which can correct misincorporated nucleotide during DNA synthesis^4^,^5^. TransHF and LATaq are polymerase blend composed of Taq or KlenTaq (an N-terminal deleted version of Taq) and a small portion of proofreading DNA polymerase. The polymerase blend is very powerful for PCR amplifying of long DNA fragment^6^,^7^. Phusion, Q5 and Cobuddy are proofreading polymerases fused with the DNA binding protein Sso7d. Fusing with Sso7d greatly increased the processivity and fidelity of the proofreading DNA polymerases such as Pfu, and enables them to PCR amplify long DNA fragment^8^. The modification for engineering PfuFly is not disclosed. PSGXL is a mutated form of PS and enhanced by a processivity-enhancing factor. As TaKaRa revealed that PSGXL is not an Sso7d-fused polymerase, probably it is enhanced by proliferating cell nuclear antigen, a processivity-enhancing protein complex^5^.

### PCR conditions

PCR primer information is listed in Table 1. PCR programs for Phusion, Q5, Cobuddy, PSGXL and FastPfuFly is: initial denaturation at 98 °C for 30 s; followed by 25–35 cycles of denaturation at 98 °C for 10 s, and annealing and extension at 66 °C for 30 s/kilobase (kb); and a final extension at 66 °C for 5 min. The same programs except that annealing and extension for 50 s/kb and 2 min/kb were used for LATaq and Pfu, respectively. For Taq and TransHF, the initial denaturation was at 94 °C for 3 min, and the cycling was 94 °C for 30 s, and 66 °C for 50 s/kb. All PCRs were carried out in a total volume of 25 μl. Primers were used at a final concentration of 0.4 μM unless specified. Each 50 ng of mouse genomic DNA, 1 ng of λ phage DNA, 0.1 ng of pBlueScript II KS (−), or cDNA from 20 ng of mouse brain total RNA was used as template. A homemade GC enhancer consisting of 2.5 M of betaine, 1 M of trehalose and 12.5% (v/v) of DMSO was used at various concentrations to enhance PCR performance. The GC enhancer was also used as a 5X stock solution in reverse transcription to improve cDNA synthesis.

### Electrophoresis mobility shift assay (EMSA)

EMSA was performed using LightShift™ Chemiluminescent EMSA Kit (Thermo Fisher Scientific Inc.). For binding, 50 fmol of 5′-Biotin labeled oligonucleotide Bio-G6 (please see Table 1 for sequences of Bio-G6, G6 and MG) was mixed with 0.75 U of Taq, Phusion or PSGXL in1X Binding Buffer, and 50 pmol of unlabeled G6 and MG was used as specific competitor and non-specific competitor, respectively. After 20 min incubation at room temperature, the samples were mixed with 5X Loading Buffer and loaded onto 6% polyacrylamide gel in 0.5X TBE. Oligonucleotides were electrophoresed and transferred onto Hybond-NX membrane (GE Healthcare). Chemiluminescent detection was performed according to the user manual of LightShift™ Chemiluminescent EMSA Kit.

## Additional Information

**How to cite this article**: Zhu, X.-J. *et al*. Guanine-rich sequences inhibit proofreading DNA polymerases. *Sci. Rep.* **6**, 28769; doi: 10.1038/srep28769 (2016).

## Figures and Tables

**Table 1:**
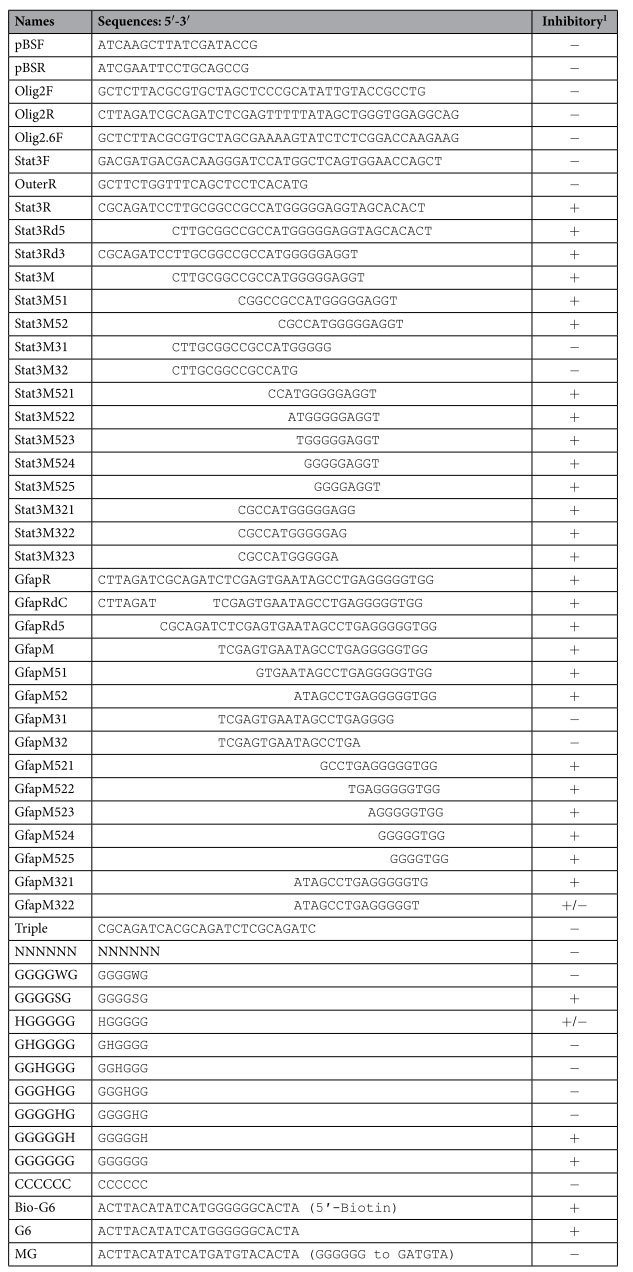
Information of oligonucleotides.

Please see Fig. 3 for their inhibitory effect. +: strong inhibition. +/−: mild inhibition. −: no inhibition.
